# The Oral Microbiome and Its Role in Systemic Autoimmune Diseases: A Systematic Review of Big Data Analysis

**DOI:** 10.3389/fdata.2022.927520

**Published:** 2022-06-29

**Authors:** Lu Gao, Zijian Cheng, Fudong Zhu, Chunsheng Bi, Qiongling Shi, Xiaoyan Chen

**Affiliations:** ^1^Stomatology Hospital, School of Stomatology, Zhejiang University School of Medicine, Hangzhou, China; ^2^Zhejiang Provincial Clinical Research Center for Oral Diseases, Hangzhou, China; ^3^Key Laboratory of Oral Biomedical Research of Zhejiang Province, Cancer Center of Zhejiang University, Hangzhou, China

**Keywords:** oral microbiome, systemic autoimmune disease, systemic lupus erythematosus, rheumatoid arthritis, Sjögren's syndrome, high-throughput analysis

## Abstract

**Introduction:**

Despite decades of research, systemic autoimmune diseases (SADs) continue to be a major global health concern and the etiology of these diseases is still not clear. To date, with the development of high-throughput techniques, increasing evidence indicated a key role of oral microbiome in the pathogenesis of SADs, and the alterations of oral microbiome may contribute to the disease emergence or evolution. This review is to present the latest knowledge on the relationship between the oral microbiome and SADs, focusing on the multiomics data generated from a large set of samples.

**Methodology:**

By searching the PubMed and Embase databases, studies that investigated the oral microbiome of SADs, including systemic lupus erythematosus (SLE), rheumatoid arthritis (RA), and Sjögren's syndrome (SS), were systematically reviewed according to the PRISMA guidelines.

**Results:**

One thousand and thirty-eight studies were found, and 25 studies were included: three referred to SLE, 12 referred to RA, nine referred to SS, and one to both SLE and SS. The 16S rRNA sequencing was the most frequent technique used. HOMD was the most common database aligned to and QIIME was the most popular pipeline for downstream analysis. Alterations in bacterial composition and population have been found in the oral samples of patients with SAD compared with the healthy controls. Results regarding candidate pathogens were not always in accordance, but *Selenomonas* and *Veillonella* were found significantly increased in three SADs, and *Streptococcus* was significantly decreased in the SADs compared with controls.

**Conclusion:**

A large amount of sequencing data was collected from patients with SAD and controls in this systematic review. Oral microbial dysbiosis had been identified in these SADs, although the dysbiosis features were different among studies. There was a lack of standardized study methodology for each study from the inclusion criteria, sample type, sequencing platform, and referred database to downstream analysis pipeline and cutoff. Besides the genomics, transcriptomics, proteomics, and metabolomics technology should be used to investigate the oral microbiome of patients with SADs and also the at-risk individuals of disease development, which may provide us with a better understanding of the etiology of SADs and promote the development of the novel therapies.

## Introduction

Autoimmune diseases are a heterogeneous group of multifactorial disorders characterized by abnormal immune responses to the body's own cells or tissues (Bolon, 2012). Generally, the immune system can distinguish foreign pathogens from the body's own cells and tissues and thus does not respond to the biomolecules expressed in endogenous tissues, which is so called “self-tolerance” (Ahsan, 2017). When the self-tolerance is damaged, the immune system will produce autoantibodies binding to the target tissues and cause destruction (Xiao et al., 2021). Autoimmune diseases can be classified into organ-specific and systemic autoimmune diseases based on the range of tissues targeted by autoantibodies (Inanç, 2020). The common systemic autoimmune diseases (SADs) include rheumatoid arthritis (RA), systemic lupus erythematosus (SLE), and Sjögren's Syndrome (SS), affecting more than 5% of people worldwide (Van Loveren et al., 2001), women predominantly (Credendino et al., 2020; Willame et al., 2021). SADs can cause chronic, systemic, excessive immune response and inflammation, resulting in a series of mild to life-threatening symptoms, such as fatigue, dizziness, malaise, fever, neurological problems, anemia, and thrombocytopenia (Wang et al., 2015). Although the symptoms can be managed by the treatment, there are no cures for SADs currently. Treatment depends on the type of disease but often includes immune suppression, which can lead to compromised immunity and vulnerability to other diseases after long-term use (Ostrov, 2015). Although a complex interplay of variable genetic risks, environmental factors, and hormonal factors is thought to contribute to breaking the immunological tolerance, the etiology of SADs remain undefined, and more effective therapies are needed (Wahren-Herlenius and Dörner, 2013).

Autoimmunity develops in the context of the human microbiome, which is defined as the full complement of microorganisms and its collective genetic materials at a particular location (Ursell et al., 2012). Inside the human body, the oral microbiome is considered to be the second largest and diverse microbiome following the gut microbiome (Verma et al., 2018). The oral microbiome comprises billions of microorganisms composed of more than 700 species of bacteria, as well as fungi, viruses, and protozoa (Deo and Deshmukh, 2019). The oral microbiome can have an impact on the general health of an individual (Lamont et al., 2018). Periodontitis, a microbially-induced inflammatory condition that causes damage to the supporting tissues of the teeth, alongside its related pathogens, may be a risk factor for cardiovascular diseases (Tonetti and Van Dyke, 2013), preterm or low birth weight babies (Teshome and Yitayeh, 2016), rheumatoid arthritis (de Molon et al., 2019), or diabetes (Sanz et al., 2018). Oral bacteria can act as opportunistic pathogens at distant sites in the body, e.g., following entry to the bloodstream (bacteraemia) or aspiration into the lungs (Potgieter et al., 2015).

To date, with the development of high-throughput techniques and the availability of multi-omics data generated from a large set of samples, increasing studies have tried to investigate the link between microbiome and SADs, suggesting that perturbations of the oral microbiome may influence the emergence or evolution of autoimmunity (Chu et al., 2021; Doaré et al., 2021). However, it is undefined whether the oral microbial dysbiosis is a consequence of bad oral hygiene or periodontitis. There are many different high-throughput techniques, analysis pipelines, and bioinformatics tools available to use but no agreement has been reached to set a standard methodology. Big data analysis after sequencing is also a significant challenge for researchers because it is highly computationally demanding.

The aim of this review is to present the latest knowledge on the relationship between the oral microbiome and SADs, focusing on the multi-omics data generated from a large set of samples.

## Methods

### Information Sources and Search Process

By searching the PubMed and Embase databases, systematic research was performed according to the PRISMA guidelines (Page et al., 2021). All articles published from 1 January 2000 to 1 January 2022 were taken into account. The search queries follow: [“oral” AND “microbiota” OR “microbiome” OR “dysbiosis” OR “flora”] AND [“systemic lupus erythematosus” OR “Lupus Erythematosus, Systemic” OR “Libman Sacks Disease” OR “rheumatoid arthritis” OR “Sjogren's Syndrome” OR “Sicca Syndrome” OR “SS”].

### Eligibility Criteria

To be eligible for inclusion, studies should provide the evaluation of oral microbiome (e.g., the composition and/or diversity of the oral microbial community) from oral samples (rinsing samples, subgingival dental plaque, buccal swab, saliva, etc.) in patients with SADs by multi-omics approaches.

All patients with SLE within the studies should satisfy one of the classification criteria of the American College of Rheumatology (ACR) 1982/1997 criteria (Hochberg, 1997) or the Systemic Lupus International Collaborating Clinics (SLICC) 2012 criteria (Petri et al., 2012). All patients with RA within the studies should satisfy the classification criteria of the American Rheumatism Association (ARA) 1987 criteria (Arnett et al., 1988) or the American College of Rheumatology/European League Against Rheumatism (ACR/EULAR) 2010 criteria (Aletaha et al., 2010). All patients with SS should satisfy the classification criteria of the ACR/EULAR 2016 criteria (Shiboski et al., 2017) or the American-European Consensus Group (AECG) 2002 criteria (Vitali et al., 2002) or the ACR 2012 criteria (Shiboski et al., 2012).

Studies were excluded if they (1) did not clarify the diagnosis criteria; (2) included patients secondary to other diseases; (3) only evaluated the oral microbiome by bacterial culture or DNA hybridization technology; (4) only evaluated the gut microbiome; (5) were reviews; (6) were not written in English; (7) were *in vitro* studies.

### Study Selection

The studies were selected by two authors (L.G. and ZJ.C.) based on the inclusion/exclusion criteria and by considering titles and abstracts, with any disputes resolved by a third author (CS.B.). Then the authors analyzed the full-text selected studies again and determined the eligible articles.

### Data Collection

Standardized extraction was used to extract the features of the included studies. The following data were extracted: (1) oral sample type, (2) region, (3) sample size, (4) confounding variables, (5) dental status, (6) use of antibiotics, (7) sequencing platform, (8) pipeline for data analysis, (9) referred database, and (10) specific changes in the oral microbiome associated with SADs.

## Results

### Study Search

**Figure 1:**
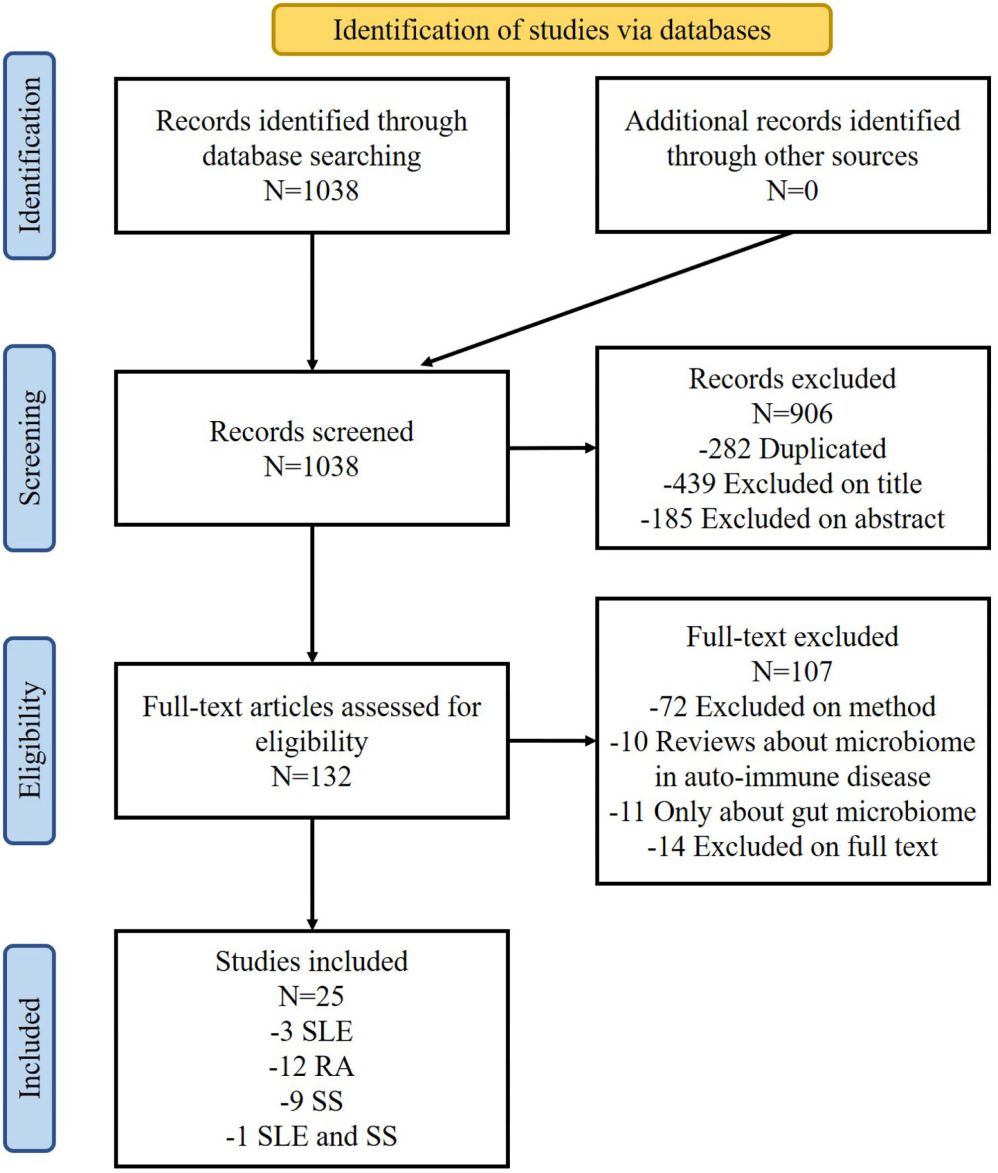
Flow-chart diagram of the selection process.

One thousand and thirty-eight studies were identified from the Embase and PubMed databases. Duplicate references (*n* = 282) were removed and 624 were excluded by title and abstract. Of the remaining 132 studies, 107 were excluded through full-text selection. A total of 25 studies were finally included and their data were extracted. Among these, three studies were referred to the SLE, 12 referred to RA, nine referred to SS, and one study referred to both SLE and SS (Figure 1).

### General Population Characteristics

In total, 137 SLE, 760 RA, and 189 primary SS (pSS) patients were included with information. The control group consisted of healthy volunteers free of any autoimmunity diseases for most studies (22/25) (Table 1). In addition, patients with osteoarthritis (OA) (Chen et al., 2018; Mikuls et al., 2018) and at-risk individuals of RA development who have no clinical symptoms of RA (Tong et al., 2019; Cheng et al., 2021; Kroese et al., 2021) were included for comparison with RA patients. Non-SS sicca patients were compared with SS patients (van der Meulen et al., 2018a; Rusthen et al., 2019; Alam et al., 2020).

**Table 1:** List of the general population characteristics.

**Disease**	**References**	**Oral sample type**	**Region**	**Sample size**	**Confounding variables**	**Dental status**
SLE	Liu et al., 2021	Saliva	Asia	35 SLE 35 HCs	No antibiotics Sex- and age-matched SLE group currently receives low-dose prednisone and hydroxychloroquine	Without oral disease
SLE	Li et al., 2020	Buccal swab	Asia	20 SLE 19 HCs	Similar age, BMI and diet	No data
SLE	van der Meulen et al., 2019	Oral washings Buccal swab	Europe	30 SLE 39 SS 965 HCs	Similar age, sex, ethnic background, BMI and smoking status SS vs. SLE HCs were not matched to SS or SLE patients	No data
SLE	Corrêa et al., 2017	Subgingival dental plaque	America	52 SLE (17 NCP and 35 CP) 52 non-SLE (24 NCP and 28 CP)	Similar age, sex and oral hygiene habits no difference in smoking status	PD, CAL, BOP, PI, TL
RA	Esberg et al., 2021	Saliva	America	61 eRA 59 HCs	No antibiotics Similar gender and age	PD, TL
RA	Kroese et al., 2021	Tongue Saliva Subgingival dental plaque	Europe	50 eRA 50 at-risk of RA 50 HCs	Gender- and age- matched Similar smoking status, alcohol consumption, use of drugs, use of antibiotics within 3 months and oral hygiene status	PD, BOP, PISA
RA	Cheng et al., 2021	Subgingival dental plaque	Europe	26 eRA 48 at-risk of RA 32 HCs	No antibiotics Balanced for age, gender, and smoking status	PD, CAL, BOP, PI, TL
RA	Lehenaff et al., 2021	Subgingival dental plaque from shallow and deep sites	America	8 RA 10 household members of the RA patients	No antibiotics Similar age, gender, race, number of caries, and periodontal health status	CAL, PD, BOP
RA	de Jesus et al., 2021	Buccal swab	America	35 RA 64 non-RA	No antibiotics Similar oral health status, smoking status	Self-reported oral health symptoms denture, gum bleeding
RA	Tong et al., 2019	Saliva	Asia	27 RA 29 at-risk of RA 23 HCs	No antibiotics Similar age, gender, and smoking status	Self-reported questionnaire
RA	Corrêa et al., 2019	Subgingival dental plaque	America	42 RA (21 CP and 21 NCP) 47 HCs (20 CP and 27 NCP)	No antibiotics Gender- and age- matched Similar smoking status	PD, CAL, BOP, PI
RA	Mikuls et al., 2018	Subgingival dental plaque	America	260 RA 296 OA	No antibiotics Similar age, gender and race	Full mouth periodontal evaluation
RA	Lopez-Oliva et al., 2018	Subgingival dental plaque	Europe	22 RA 19 HCs (both periodontally healthy)	No antibiotics Similar gender, race, smoking history and alcohol consumption	CAL, PD, BOP
RA	Chen et al., 2018	Saliva	Asia	110 RA 67 OA 155 HCs	Gender and age not matched	No data
RA	Zhang et al., 2015	Dental plaque	Asia	54 RA 51 HCs	No antibiotics Age-, gender-, and ethnicity-matched	No data
		Saliva		51 RA 47 HCs		
RA	Scher et al., 2012	Subgingival dental plaque	America	31 NORA 34 CRA 18 HCs	No antibiotics Age-, gender-, and ethnicity-matched	CAL, PD, BOP
SS	Sharma et al., 2020	Saliva	Asia	37 SS 35 HCs	No antibiotics No smoking Similar gender	No data
SS	Alam et al., 2020	Oral washings	Asia	8 SS without oral dryness 17 SS with dryness 11 sicca 14 HCs	No smoking, no antibiotics and steroids Similar gender, age	No data
SS	Rusthen et al., 2019	Saliva	Europe	15 SS 15 sicca 15 HCs	Similar gender, age, smoking and dental status	Missing and decayed teeth, number of mobile teeth and gingivitis, dental caries experience
SS	Sembler-Møller et al., 2019	Saliva	Europe	24 SS 34 sicca	No smoking, no antibiotics Similar age, gender, general health, oral health status	DMFT and DMFS, dental plaque, gingival inflammation and periodontal pocket depth
SS	Zhou et al., 2018	Oral washings	Asia	22 SS 23 HCs	Similar gender and age	DMFT and DMFS
SS	van der Meulen et al., 2018a	Buccal swab	Europe	37 SS 86 sicca 24 HCs	Gender matched Age not matched	Own teeth, oral dryness
SS	de Paiva et al., 2016	Tongue	America	10 SS 11 HCs	Similar gender and age	No data
SS	Siddiqui et al., 2016	Saliva	Europe	9 SS 9 HCs	Similar gender and age No hyposalivation	No data
SS	Li et al., 2016	Buccal swab	Asia	10 SS 10 HCs	No smoking, no antibiotics Similar gender and age, number of teeth, periodontal and mucosal status	Oral mucosa, number of teeth and stimulated/ unstimulated secretion rat

*SLE, systemic lupus erythematosus; RA, rheumatoid arthritis; SS, Sjögren's syndrome; HCs, healthy controls; NCP, non-chronic periodontitis; CP, chronic periodontitis; OA, osteoarthritis; NORA, new-onset rheumatoid arthritis, disease duration of >6 weeks and absence of any treatment with disease-modifying anti-rheumatic drug (DMARD) or steroids (ever); eRA, early onset of RA, symptom duration ≤ 12 months; CRA, chronic RA with minimum disease duration of 6 months; PD, probing depth; CAL, clinical attachment level; BOP, bleeding on probing; PI, plaque index; PISA, periodontal inflamed surface area; TL, tooth loss; DMFT, decayed, missing and filled teeth; DMFS, decayed, missing and filled surfaces*.

Most studies considered gender (22/25), age (20/25), smoking status (11/25), use of antibiotics (14/25), and dental status (17/25) as confounding variables. The exclusion criteria about the use of antibiotics varied from 2 to 12 weeks before the sample collection.

Although 68% studies (17/25) (Table 1) took the dental status into consideration, the method of dental assessment was different across studies. Two studies used the self-reported symptoms for assessment (Tong et al., 2019; de Jesus et al., 2021). Nine studies provided a full periodontal examination to assess the parameters including probing depth (PD), clinical attachment level (CAL), and bleeding on probing (BOP). Three studies performed a detailed caries-related registration on decayed, missing, and filled teeth/-surfaces (DMFT/DMFS; Zhou et al., 2018; Rusthen et al., 2019; Sembler-Møller et al., 2019). However, the information about dental treatment was not always considered. Only two studies claimed that the volunteers were free of treatment for periodontal disease within the last 6 months (Corrêa et al., 2017, 2019).

The oral sample type differed between studies (Table 1). Saliva was collected in 10 studies and subgingival dental plaque was collected in nine studies using sterile paper points. Oral washings, sterile cotton swabs on buccal mucosa, and dorsum of the tongue were also employed.

All the individuals included in each study were local residents (Table 1). Among them, nine studies analyzed the oral microbiome of Asians, of which 77.8% (7/9) studies referred to Chinese people. Eight studies investigated the oral microbiome of Europeans and the other eight studies focused on Americans.

### General Analysis Characteristics

The most common analysis method was 16S rRNA gene sequencing, which was used in 92% of studies (23/25) (Table 2). Only two studies (Zhang et al., 2015; Cheng et al., 2021) used a shotgun metagenomics approach to investigate the oral microbiome of patients with RA. The Human Oral Microbiome Database (HOMD) was the most popular database used for taxonomic assignment, although the similarity threshold was different between studies ranging from 95 to 100% identity. Most 16S rRNA gene sequencing analyses (13/23) were performed with at least 97% similarity when clustering the sequences for operational taxonomic unit (OTU), while the shotgun metagenomics used a less stringent cutoff (95%) instead (Table 2). QIIME was the most widely used pipeline (16/25) for the downstream analysis and sometimes was used along with other software such as Mothur, PhyloToAST, and LoTuS.

**Table 2:** Analysis of the methodology of the included studies.

**Disease**	**References**	**Sequencing platform**	**Database**	**Pipeline**	**Identity**
SLE	Liu et al., 2021	16S rRNA sequencing/Illlumina MiSeq platform	Greengenes V.13-8	QIIME 2	99%
SLE	Li et al., 2020	16S rRNA sequencing	SILVA 128 database	Mothur	97%
SLE	van der Meulen et al., 2019	16S rRNA sequencing	SILVA 128 database	QIIME	No data
SLE	Corrêa et al., 2017	16S rRNA sequencing/Illumina MiSeq platform	CORE	QIIME	No data
RA	Esberg et al., 2021	16S rRNA sequencing/Illlumina MiSeq platform	eHOMD	QIIME 2	>98.5%
RA	Kroese et al., 2021	16S rDNA sequencing/Illumina MiSeq platform	HOMD	QIIME v1.8.0	No data
RA	Cheng et al., 2021	Shotgun metagenomics sequencing/Illumina HiSeq 3000 platform	MG-RAST	Refseq	95%
RA	Lehenaff et al., 2021	16S rRNA sequencing/Illumina Miseq platform	HOMD	QIIME2	97%
RA	de Jesus et al., 2021	16S rRNA sequencing/Illumina Miseq PE250 platform	HOMD	QIIME2	No data
RA	Tong et al., 2019	16S rRNA sequencing/Illumina Miseq platform	SILVA 128 database	/	97%
RA	Corrêa et al., 2019	16S rRNA sequencing/Illumina MiSeq platform	CORE	QIIME	97%
RA	Mikuls et al., 2018	16S rRNA sequencing/Illumina MiSeq platform	HOMD	/	97%
RA	Lopez-Oliva et al., 2018	16S rRNA sequencing/Illumina MiSeq platform	HOMD	QIIME PhyloToAST	97%
RA	Chen et al., 2018	16S rRNA sequencing/HiSeq 2500 platform	Greengenes ribosomal database	QIIME 1.9.1	97%
RA	Zhang et al., 2015	Metagenomic shotgun sequencing and a metagenome-wide association study (MGWAS)/Illumina platform	Microbial Genomes (IMG, v400) database	in-house pipeline	95%
RA	Scher et al., 2012	16S rRNA sequencing/454 GS FLX Titanium platform	SILVA 128 database	Mothur	97%
SS	Sharma et al., 2020	16S rRNA sequencing/HiSeq 2500 platform	Greengene database SILVA 128 database	QIIME LoTuS	97%
SS	Alam et al., 2020	16S rRNA sequencing/454 GS FLX titanium pyrosequencer	The EzTaxon-e database	No data	No data
SS	Rusthen et al., 2019	16S rRNA sequencing/Roche 454 GS Junior platform	SILVA 128 database HOMD	QIIME 1.8.0	99–100%
SS	Sembler-Møller et al., 2019	16S rRNA sequencing/Illumina Miseq platform	eHOMD	DADA2 R	No data
SS	Zhou et al., 2018	16S rRNA sequencing/Illumina Miseq PE300 platform	HOMD	Mothur QIIME 1.9.1	97%
SS	van der Meulen et al., 2018a	16S rRNA sequencing/Illumina MiSeq platform	HOMD	QIIME V.1.9.1	97%
SS	de Paiva et al., 2016	16S rRNA sequencing/MiSeq platform	UPARSE and the SILVA 128 database	No data	97%
SS	Siddiqui et al., 2016	16S rRNA sequencing/454 GS Junior system	HOMDEXTGG set the NCBI 16S rRNA reference sequence set	QIIME 1.9.1	98%
SS	Li et al., 2016	16S rRNA sequencing/NGS illumine Miseq 2 × 300 bp platform	SILVA dataset	Mothur	97%

*SLE, systemic lupus erythematosus; RA, rheumatoid arthritis; SS, Sjögren's syndrome; HOMD, Human Oral Microbiome Database; eHOMD, expanded Human Oral Microbiome Database*.

### Oral Microbial Dysbiosis Features

Oral microbial dysbiosis has been identified in the three SADs included in our review, although inconsistent results exist (Tables 3–**6**). Sembler-Møller et al. (2019) reported that there was no significant difference in the oral bacterial diversity or relative abundance on the genus and species level between SS and non-SS controls, indicating that changes in the salivary microbiome was not related to the SS itself.

**Table 3:** Major changes in microbial community associated with SADs.

**Disease**	**References**	**Oral sample type**	**Alpha diversity**	**Beta diversity**
SLE	Liu et al., 2021	Saliva	No significant change	Increased bacterial diversity in SLE patients compared with HCs
SLE	Li et al., 2020	Buccal swab	Lower alpha- diversity in SLE patients compared with HCs	Higher beta- diversity in SLE patients compared with HCs
SLE	van der Meulen et al., 2019	Oral washings Buccal swab	Higher alpha- and beta- diversity in SLE patients compared with SS patients	
SLE	Corrêa et al., 2017	Subgingival dental plaque	Higher alpha-diversity in SLE patients compared with HCs	Lower beta-diversity in SLE patients compared with HCs
RA	Esberg et al., 2021	Saliva	Higher alpha- and beta- diversity in SLE patients compared with HCs	
RA	Kroese et al., 2021	Tongue Saliva Subgingival dental plaque	/	/
RA	Cheng et al., 2021	Subgingival dental plaque	Lower richness and diversity in CCP+ at-risk group and the eRA group compared with HCs	
RA	Lehenaff et al., 2021	Subgingival dental plaque	No significant difference between RA patients and HCs	
RA	de Jesus et al., 2021	Buccal swab	Similar Shannon diversity index of bacterial species among RA compared with non-RA controls	Significant difference between RA and controls
RA	Tong et al., 2019	Saliva	Lower alpha- diversity in high-risk group compared with HCs	A tendency of gradual lower change from HCs, high-risk group to RA patients
RA	Corrêa et al., 2019	Subgingival dental plaque	Higher bacterial richness than controls without periodontitis	Increased microbial diversity compared with controls
RA	Mikuls et al., 2018	Subgingival dental plaque	No difference between RA and OA patients	/
RA	Lopez-Oliva et al., 2018	Subgingival dental plaque	/	/
RA	Chen et al., 2018	Saliva	Higher diversity in RA and OA compared with HCs, but no difference between RA and OA	Higher diversity in RA and OA compared with HCs, lower diversity in RA compared with OA
RA	Zhang et al., 2015	Dental plaque Saliva	Increased richness and diversity in RA patients compared with HCs	
RA	Scher et al., 2012	Subgingival dental plaque	The oral microbiota is equally rich and diverse in NORA, CRA and control groups	
SS	Sharma et al., 2020	Saliva	No difference between SS patients and HCs	
SS	Alam et al., 2020	Oral washings	Higher diversity in SS patients compared with HCs	/
SS	Rusthen et al., 2019	Saliva	No difference in SS, sicca and HCs	
SS	Sembler-Møller et al., 2019	Saliva	No difference between SS and sicca	
SS	Zhou et al., 2018	Oral washings	Lower oral bacterial community evenness and diversity in SS patients compared with HCs	No difference between SS and HCs
SS	van der Meulen et al., 2018a	Buccal swab	No difference among SS, sicca and HCs, but showed a trend towards lower richness and diversity compared with HCs	
SS	de Paiva et al., 2016	Tongue	Lower Shannon diversity in SS compared with HCs	/
SS	Siddiqui et al., 2016	Saliva	Lower species richness, alpha- diversity in SS compared with HCs	/
SS	Li et al., 2016	Buccal swab	No difference between SS patients and HCs	

*SLE, systemic lupus erythematosus; RA, rheumatoid arthritis; SS, Sjögren's syndrome; HCs, healthy controls; OA, osteoarthritis; eRA, early onset of RA, symptom duration ≤ 12 months; NORA, new-onset rheumatoid arthritis, disease duration of >6 weeks and absence of any treatment with disease-modifying anti-rheumatic drug (DMARD) or steroids (ever); CRA, chronic RA with minimum disease duration of 6 months*.

**Figure 2:**
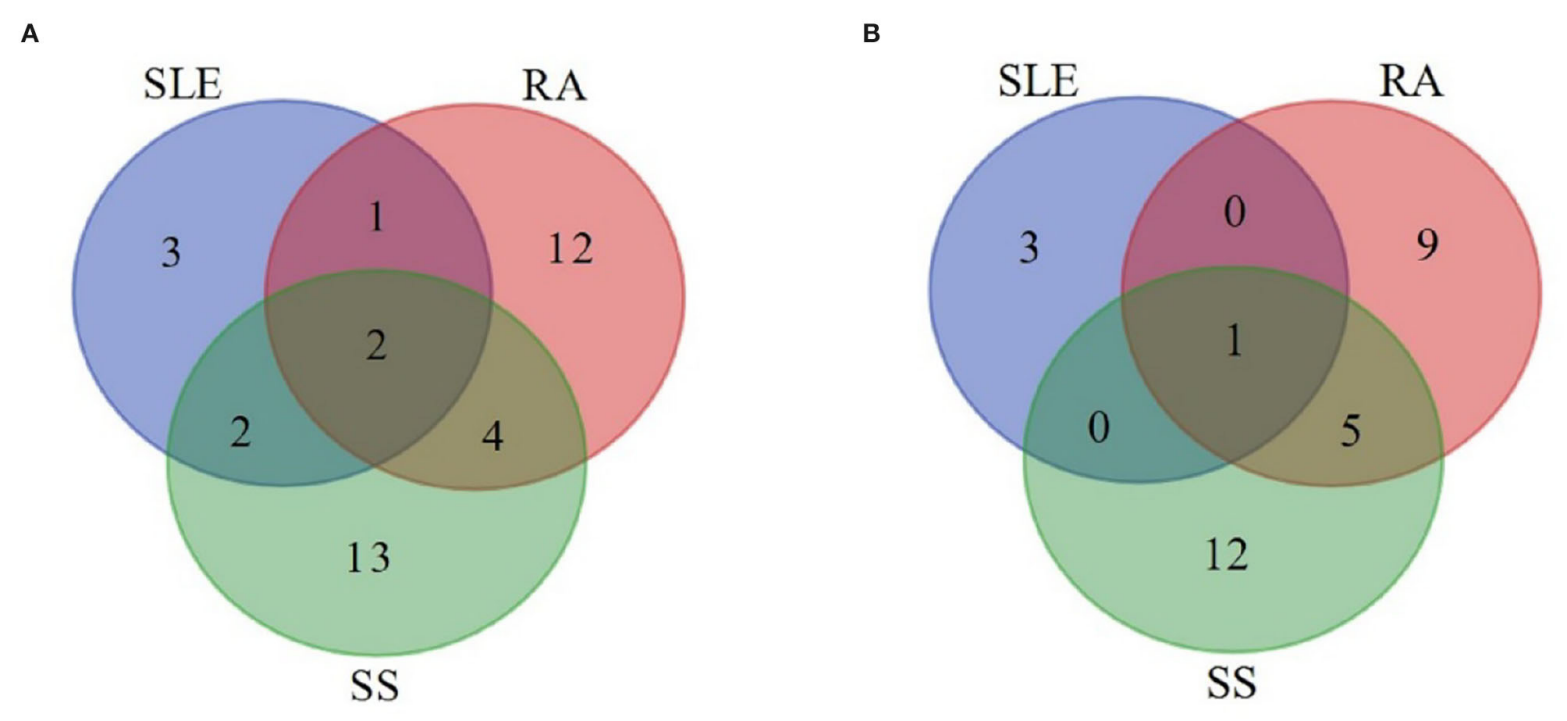
Overlap analysis of the significantly increased **(A)** and decreased **(B)** genera in systemic autoimmune diseases. Numbers of the increased **(A)** and decreased **(B)** genera were visualized for SLE, RA, and SS patients. SLE, systemic lupus erythematosus; RA, rheumatoid arthritis; SS, Sjögren's syndrome.

**Figure 3:**
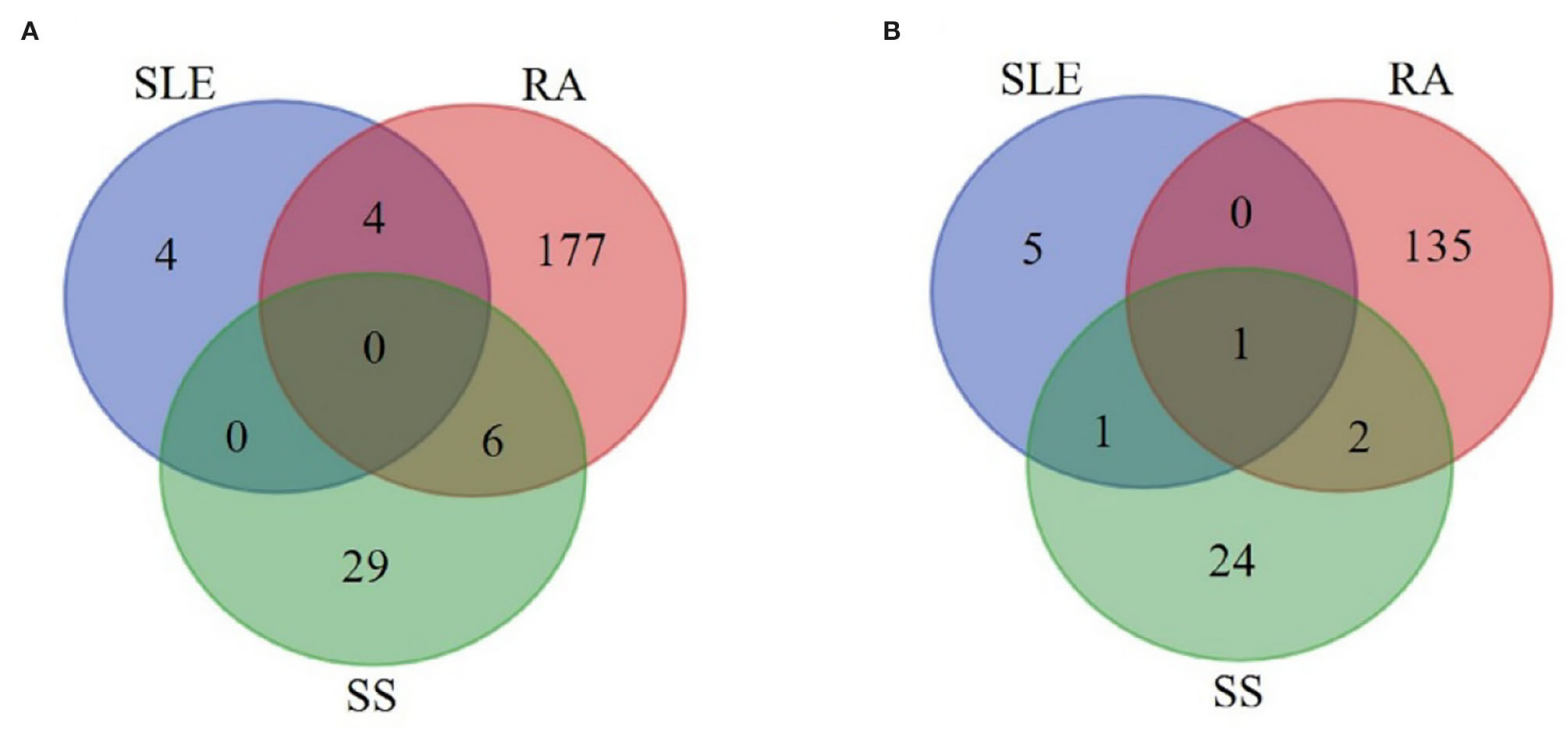
Overlap analysis of the significantly increased **(A)** and decreased **(B)** species in systemic autoimmune diseases. Numbers of the increased **(A)** and decreased **(B)** species were visualized for SLE, RA, and SS patients. SLE, systemic lupus erythematosus; RA, rheumatoid arthritis; SS, Sjögren's syndrome.

Among the 25 articles included, *Selenomonas* and *Veillonella* were found significantly increased in the three SADs covered by this review, and *Streptococcus* was significantly decreased in the SADs compared with controls (Figure 2). At the species level (Figure 3), *Rothia aeria* was significantly decreased in all three diseases. *Prevotella nigrescens, Prevotella oulorum, Prevotella pleuritidis*, and *Selenomonas noxia* were identified enriched in both RA and SLE compared with healthy controls. *Prevotella salivae, Prevotella histicola, Lactobacillus salivarius, Prevotella melaninogenica, Streptococcus parasanguinis*, and *Porphyromonas endodontalis* were more abundant in patients with RA and SS.

#### Systemic Lupus Erythematosus

The oral microbial dysbiosis features in the SLE patients are summarized in Tables 3, 4.

**Table 4:** Specific changes in the oral microbiome of SLE patients.

**References**	**Enriched genus**	**Decreased genus**	**Enriched species**	**Decreased species**
Liu et al. (2021)	*Prevotella, Selenomonas*, and *Veillonella*	*Bacteroides* and *Streptococcus*	/	/
Li et al. (2020)	*Barnesiella, Blautia, Lactobacillus, Pyramidobacter* and *Veillonella*	/	/	/
van der Meulen et al. (2019)	**SLE vs. HCs:** *Alistipes*	/	/	/
Corrêa et al. (2017)	/	**In NCP group:** *Sphingomonas* **In CP group:** *Clostridiales*	**In NCP group:** *Prevotella* (*P. nigrescens, P. oulorum, P. oris*), and *Selenomonas noxia* **In CP group:** *Prevotella* (*P. oulorum, P. pleuritidis*), *Pseudomonas spp., Treponema maltophilum* and *Actinomyces* IP073	**In CP group:** *Rothia aeria, Capnocytophaga gingivalis, Rasltonia* oral taxon 027, *Leptotrichia* oral taxon A71, *Streptococcus sanguinis* and *Haemophilus parainfluenzae*

*SLE, systemic lupus erythematosus; NCP, non-chronic periodontitis; CP, chronic periodontitis*.

With regard to the studies about the oral microbiome in patients with SLE, all the four studies assessed alpha- and beta- diversity and found that there were significant differences between the SLE patients and controls (Table 3). But the results were not consistent among the studies, which may be due to the different sample types relied on. One study analyzing the subgingival dental plaque found higher alpha diversity in patients with SLE compared with healthy controls (Corrêa et al., 2017), while another study focusing on the buccal swabs found decreased bacterial diversity in patients with SLE compared with healthy controls (Li et al., 2020).

As shown in Table 4, *Veillonella, Prevotella, Selenomonas, Blautia, Barnesiella, Pyramidobacter, Alistipes*, and *Lactobacillus* were more abundant in patients with SLE compared with healthy controls when analyzing the oral microbiome at the genus level. There was only one study that analyzed the subgingival dental plaque of patients with SLE and presented changes in subgingival microbiome at the species level (Corrêa et al., 2017). By periodontal assessment of the participants, species associated with SLE had been identified in the non-periodontitis group. *Prevotella nigrescens, Prevotella oulorum, Prevotella oris*, and *Selenomonas noxia* were more abundant in the patients with SLE compared with healthy controls. The results of this study indicated that oral microbial dysbiosis was associated with SLE, independent of periodontal status.

#### Rheumatoid Arthritis

The oral microbial dysbiosis features in the patients with RA are summarized in Tables 3, 5.

**Table 5:** Specific changes in the oral microbiome of RA patients.

**References**	**Enriched genus**	**Decreased genus**	**Enriched species**	**Decreased species**
Esberg et al. (2021)	/	/	*Prevotella pleuritidis, Porphyromonas endodontalis, Filifactor alocis* and *Treponema denticola*	*Oribacterium sinus, Catonella morbi, Veillonella rogosae* and *Campylobacter concisus*
Kroese et al. (2021)	*Veillonella, Prevotella*	/	*Prevotella salivae*	*Neisseria flavescens, Streptococcus dentisani, Porphyromonas pasteri* and *Veillonella parvula*
Cheng et al. (2021)	**Periodontally healthy site** *Cardiobacterium, Bifidobacterium, Porphyromonas, Capnocytophaga, Neisseria* and *Streptococcus* **Diseased site** *Cardiobacterium, Capnocytophaga, Neisseria* and *Streptococcus*	/	**Periodontally healthy site:** *Acinetobacter baumannii, Acinetobacter johnsonii, Acinetobacter lwoffii, Alistipes putredinis, Cardiobacterium hominis, Caulobacter segnis, Clostridium phytofermentans, Enhydrobacter aerosaccus, Enterococcus casseliflavus, Methylobacterium extorquens, Methylobacterium nodulans, Methylobacterium populi, Methylobacterium radiotolerans, Pseudomonas stutzeri, Shewanella sp. ANA-3, Sphingopyxis alaskensis, Thiomonas intermedia, Xanthobacter autotrophicus* and *Xanthomonas campestris* **Diseased site:** *Capnocytophaga gingivalis, Cardiobacterium hominis, Eikenella corrodens, Neisseria gonorrhoeae, Neisseria mucosa, Neisseria sicca, Neisseria subflava, Streptococcus mitis, Streptococcus oralis, Streptococcus pneumoniae, Streptococcus sanguinis* and *Streptococcus sp. M143*	/
Lehenaff et al. (2021)	/	/	*Actinomyces meyeri* and *Streptococcus parasanguinis*	*Gemella morbillorum, Kingella denitrificans, Prevotella melaninogenica and Leptotrichia spp*.
de Jesus et al. (2021)	*Streptococcus, Rothia* and *Leptotrichia*	*Fusobacterium, Porphyromonas, Aggregatibacter* and *Capnocytophaga*	*Streptococcus salivarius, Rothia mucilaginosa, Prevotella spp*., *Leptotrichia spp*. and *Selenomonas fueggei*	*Prevotella melaninogenica, Fusobacterium periodonticum, Granulicatella elegan*s and *Porphyromonas endodontalis*
Tong et al. (2019)	**RA vs. HCs:** *Prevotella*_6 and *Selenomonas*_3 **RA vs. at-risk:** *Rothia*	**RA vs. HCs:** *Neisseria, Haemophilus*, and *Parvimonas* **RA vs. at-risk:** *Filifactor*	/	**RA and at-risk vs. HCs:** *Defluviitaleaceae UCG-011* and *Neisseria oralis*
Corrêa et al. (2019)	*Prevotella*	*Streptococcus, Haemophilus* and *Actinomyces*	*Prevotella* (*P. melaninogenica, P. denticola, P. histicola, P. nigrescens, P. oulorum, and P. maculosa*), *Selenomonas noxia*, S. *sputigena, Anaeroglobus geminatus, Aggregaticbacter actinomycetemcomitans* and *Parvimonas micra*	*Rothia aeria* and *Kingella oralis*
Mikuls et al. (2018)	**RA vs. OA:** *Prevotella*	/	/	/
Lopez-Oliva et al. (2018)	/	/	*Actinomyces spp., Cryptobacterium spp., Dialister spp., Desulfovibrio spp., Fretibacterium spp., Leptotrichia spp., Prevotella spp., Selenomonas spp., Treponema spp*. (119), *Cryptobacterium curtum* and *Veillonellaceae* [G1]	*Aggregatibacter spp., Gemella spp., Granulicatella spp., Hemophilus spp., Neisseria spp*. and *Streptoccoci spp*. (110)
Chen et al. (2018)	**RA vs. OA:** *Neisseria, Haemophilus, Prevotella, Veillonella, Fusobacterium, Aggregatibacter* and *Actinobacillus* **RA and OA vs. HCs:** *Prevotella, Neisseria, Porphyromonas, Veillonella, Haemophilus, Rothia, Streptococcus, Actinomyces, Granulicatella, Leptotrichia, Lautropia and Fusobacterium*	**RA vs. OA:** *Streptococcus, Actinomyces, Lautropia, Rothia, Granulicatella, Ruminococcus, Oribacterium* and *Abiotrophia*	**RA vs. OA:** *Neisseria subflava, Haemophilus parainfluenzae, Veillonella dispar, Prevotella tannerae*, and *Actinobacillus parahaemolyticus* **RA and OA vs. HCs:** *Prevotella melaninogenica*, and *Veillonella dispar*	**RA vs. OA:** *Rothia dentocariosa*, and *Ruminococcus gnavus*
Zhang et al. (2015)	/	*Haemophilus, Aggregatibacter, Cardiobacterium, Eikenella* and *Kingella*	*Rothia mucilaginosa, Rothia dentocariosa, Lactobacillus salivarius, Atopobium spp*. and *Cryptobacterium curtum*	*Rothia aeria, Porphyromonas gingivalis, Lactococcus spp*., and *Cardiobacterium hominis*
Scher et al. (2012)	**NORA and CRA vs. HCs:** *Anaeroglobus, Uncl. Prevotellaceae* and *Phocaeiola*	**NORA and CRA vs. HCs:** *Corynebacterium, Mitsuokella* and *Streptococcus*	**NORA and CRA vs. HCs:** *Anaeroglobus* OTU99, *Leptotrichia* OTU87, *Prevotella* OTU60, *Selenomonas* OTU168, *Phocaeiola* OTU92, *Prevotella* OTU31, *Prevotella* OTU134, *Neisseria* OTU16, and *Porphyromonas* OTU1	**NORA and CRA vs. HCs:** *Leptotrichia* OTU12, *Leptotrichia* OTU86, *Leptotrichia* OTU9, *Capnocytophaga* OTU74, *Corynebacterium* OTU4 and *Uncl*.TM7 OTU58

*RA, rheumatoid arthritis; OA, osteoarthritis; NORA, new-onset rheumatoid arthritis, disease duration of >6 weeks and absence of any treatment with disease-modifying anti-rheumatic drug (DMARD) or steroids (ever); CRA, chronic RA with minimum disease duration of 6 months*.

Among the 11 studies that compared patients with RA with healthy controls, nine studies analyzed oral microbial diversity and richness of patients with RA, seven of which found a significant difference between patients with RA and healthy controls (Table 3), while the other two studies found no significant changes in oral microbial diversity in patients with RA (Scher et al., 2012; Lehenaff et al., 2021).

Eight studies investigated the microbiome at the genus level, and half of them found *Prevotella* significantly increased in patients with RA (Table 5). Some genera were identified with evidently different abundance in different studies. For example, by analyzing the subgingival dental plaque of patients with RA, *Streptococcus* was found with significantly higher relative abundance compared with healthy controls by Cheng et al. (2021), but was identified at a lower level in the other two studies (Corrêa et al., 2019; Tong et al., 2019). Ten studies presented the results at the species level and demonstrated different specific dysbiosis features associated with RA, of which two studies found a higher level of *Rothia mucilaginosa* in the patients with RA (Zhang et al., 2015; de Jesus et al., 2021). Two studies had performed periodontal examination on the participants, and thus were able to identify the alterations of oral microbiome in patients with RA without periodontitis (Lopez-Oliva et al., 2018; Cheng et al., 2021).

In addition, potential functions of oral microbiome were also analyzed by shotgun sequencing studies (Zhang et al., 2015; Cheng et al., 2021). Functional units were found altered in the oral microbiome of patients with RA including ATP-dependent 26S proteasome regulatory subunit, component of SCF ubiquitin ligase and anaphase-promoting complex, cysteine synthase, DNA helicase TIP49, TBP-interacting protein, serine/threonine protein phosphatase 2A, regulatory subunit, the redox environment, transport and metabolism of iron, sulfur, zinc, and arginine.

Oral microbial dysbiosis had also been discovered in the at-risk individuals of RA development, indicating that these species may be related with the RA initiation (Tong et al., 2019; Cheng et al., 2021; Kroese et al., 2021).

#### Sjögren's Syndrome

##### SS and Healthy Controls

Eight studies analyzed the alpha-diversity between patients with SS and healthy controls (Table 3). Three studies found a significantly decreased bacterial richness and alpha-diversity in patients with SS compared with healthy controls by analyzing saliva, oral washings, and tongue samples (de Paiva et al., 2016; Siddiqui et al., 2016; Zhou et al., 2018), while Alam et al. (2020) reported a significantly higher diversity in the saliva microbiome of patients with SS compared with healthy controls. Other studies found no significant differences when investigating saliva and buccal mucosa samples between the groups (Li et al., 2016; van der Meulen et al., 2018a; Rusthen et al., 2019; Sharma et al., 2020).

At genus level (Table 6), *Bifidobacterium, Lactobacillus*, and *Dialister* were found significantly increased in the saliva and buccal mucosa of patients with SS (van der Meulen et al., 2018a; Sharma et al., 2020). *Haemophilus* and *Neisseria* were found significantly decreased in four studies (Li et al., 2016; van der Meulen et al., 2018a; Zhou et al., 2018; Rusthen et al., 2019).

**Table 6:** Specific changes in the oral microbiome of SS patients.

**References**	**Enriched genus**	**Decreased genus**	**Enriched species**	**Decreased species**
Sharma et al. (2020)	*Bifidobacterium, Lactobacillus* and *Dialister*	*Leptotrichia*	/	/
Alam et al. (2020)	/	/	**SS with dryness vs. sicca:** *Veillonella parvula, Lactobacillus salivarius, Veillonella tobetsuensis, Lactobacillus fermentum* and *Veillonella rodentium* **SS vs. HCs:** *Prevotella melaninogenica, Veillonella rogosae, Streptococcus* HQ748137, *Streptococcus* HQ762034, *Prevotella histicola, Streptococcus parasanguinis, Streptococcus* 4P003152, *Streptococcus* uc, *Streptococcus mutans, Haemophilus* HQ807753, *Veillonella parvula, Prevotella* FM995711, *Streptococcus sobrinus, Prevotella salivae, Lactobacillus salivarius, Veillonella rodentium, Haemophilus haemolyticus, Lactobacillus fermentum, Prevotella* 4P003758 and *Streptococcus* HQ757980	**SS with dryness vs. sicca:** *Haemophilus sputorum, Neisseria* AY005028, *Neisseria* uc, *Capnocytophaga gingivalis, Leptotrichia wadei, Porphyromonas gingivalis, Porphyromonas* AM420091, *Lachnoanaerobaculum orale, Lautropia mirabilis, Neisseria elongata, Rothia aeria, Neisseria sicca group, Neisseria mucosa, Neisseria subflava, Streptococcus* CP006776 and *Neisseria* perflava **SS vs. HCs:** *Eikenella corrodens*
Rusthen et al. (2019)	/	**SS and sicca vs. HCs:** *Haemophilus* and *Neisseria*	**SS and sicca vs. HCs:** *Porphyromonas endodontalis, Prevotella nancensis, Tannerella spp*. and *Treponema spp*. (12) **SS vs. sicca (with hyposalivation):** *Prevotella nanceiensis*	**SS and sicca vs. HCs:** *Actinomyces lingnae, Fusobacterium nucleatum subspvincentii, Lachnoanaerobaculum orale* and *Megasphaera micronuciformis, Oribacterium asaccharolyticum, Prevotella nanceiensis, Stomatobaculum longum* and *Streptococcus intermedius* **SS vs. sicca (with hyposalivation):** *Capnocytophaga leadbetteri, Granulicatella adiacens, Neisseria flavescens*, and *RuminococcaceaeG1spt*
Sembler-Møller et al. (2019)	No significant difference	No significant difference	No significant difference	No significant difference
Zhou et al. (2018)	*Veillonella*	*Actinomyces, Haemophilus, Neisseria, Rothia, Porphyromonas* and *Peptostreptococcus*	/	/
van der Meulen et al. (2018a)	**SS vs. HCs:** *Alloscardovia, Bifidobacterium, Scardovia, Atopobium, Lactobacillus, Parvimonas, Peptostreptococcaceae, Anaeroglobus*, and *Dialister*	**SS vs. HCs:** *Alloprevotella, Bergeyella, Abiotrophia, Granulicatella, Enterococcus, Ruminococcaceae, Lautropia, Neisseria* and *Haemophilus* **SS vs. sicca:** B*ergeyella* and *Granulicatella*	/	/
de Paiva et al. (2016)	*Streptococcus*	*Leptotrichia* and *Fusobacterium*	/	/
Siddiqui et al. (2016)	*Streptococcus* and *Veillonella*	/	*Veillonella* sp. Oral Taxon 917	/
Li et al. (2016)	*Leucobacter, Delftia, Pseudochrobactrum, Ralstonia* and *Mitsuaria*	*Haemophilus, Neisseria, Comamona, Granulicatella* and *Limnohabitans*	/	/

*SS, Sjögren's syndrome; HCs, healthy controls*.

Only three studies reported results at the species level (Siddiqui et al., 2016; Rusthen et al., 2019; Alam et al., 2020). Thirty-five species, including *Streptococcus mutans, Prevotella melaninogenica*, and *Veillonella rogosae* were significantly more abundant in patients with SS compared with healthy controls (Siddiqui et al., 2016; Rusthen et al., 2019; Alam et al., 2020) and nine species were less abundant (Rusthen et al., 2019; Alam et al., 2020).

##### SS and Sicca Patients

Four studies analyzed the alpha-diversity between SS and non-SS sicca patients (Table 3). In accordance with the results of comparing SS with healthy controls, Alam et al. reported significantly a higher diversity in patients with SS compared with sicca patients (Alam et al., 2020). But others found no significant differences between patients with SS and sicca (van der Meulen et al., 2018a; Rusthen et al., 2019; Sembler-Møller et al., 2019).

At the genus level (Table 6), *Bergeyella* and *Granulicatella* were found significantly decreased in patients with SS compared with sicca patients, which were also decreased when compared with healthy controls (van der Meulen et al., 2018a). At the species level, six species were identified as significantly more abundant in patients with SS than sicca patients. Among those species, *Veillonella parvula, Lactobacillus salivarius, Lactobacillus fermentum, Prevotella nanceiensis*, and *Veillonella rodentium* were also found to be increased when comparing SS patients with healthy controls (Rusthen et al., 2019; Alam et al., 2020).

## Discussion

Currently, only symptomatic treatments are available for patients with SAD because of the unknown etiology (Zampeli et al., 2015; Fava and Petri, 2019; Ramos-Casals et al., 2020). In a healthy state, a balance is sustained between the oral microbiome and the host immune response, as well as inside the oral microbial community (Lamont et al., 2018). Therefore, the oral microbiome plays an important role in maintaining the health of the host, as well as the immune system and metabolic stability. Under the pathological conditions, the homeostasis is broken and the oral dysbiosis occurs, which usually manifests as the changes in composition and/or function of the oral microbiome (Lamont et al., 2018). Elucidating the role of the oral microbiome in the initiation and development of SADs may present new possibilities for the treatment and prevention of these diseases.

In this systematic review, we reviewed 25 studies covering 137 patients with SLE, 760 patients with RA, and 189 patients with SS with information on their oral microbiome. Oral microbial dysbiosis has been identified in the SADs in this review by comparing bacterial diversity and richness, as well as abundance of genus or species between patients and healthy controls. Significantly altered microbial diversity has been reported in patients with SLE, RA, and SS, although the inconsistent results exist, which could be due to the different sample sites of the oral cavity. Bacterial diversity of saliva microbiome, which consists mostly of gram-positive aerobes, was found elevated in patients with RA compared with controls (Chen et al., 2018; Esberg et al., 2021), while in the subgingival dental plaque that colonized predominantly by the gram-negative anaerobes or facultative anaerobes, decreased or similar diversity was reported in patients with RA compared with controls (Scher et al., 2012; Mikuls et al., 2018; Cheng et al., 2021). These findings suggested that regular periodontal maintenance or oral hygiene behavior may play an important role in the prevention and treatment of SADs. Understanding the exact association between oral microbial dysbiosis and SADs may help to develop novel combined therapies for both physicians and dentists.

*Selenomonas* and *Veillonella* were found significantly increased in the SADs covered in this review. In addition to the SADs, increased *Selenomonas* has also been identified to be associated with other systemic diseases, for example, human diabetes (Tsuzukibashi et al., 2017). Reduction of *Streptococcus*, a health-associated genus, was observed in the SADs, indicating that these SADs may disturb the oral microbiome, the mechanisms of which still need further investigation.

At the species level, significant alterations in the abundance of *Rothia aeria*, a gram-positive aerobe from the family *Micrococcaceae*, had been discovered in the three SADs, which could be explained by the abnormal immune status of those patients and also by the effect of treatment of SADs. *R. aeria* is a part of the normal human oral microbiome occasionally related with periodontal and dental infections, but has also been reported in osteomyelitis, endocarditis, and joint infections (Graves et al., 2019).

In the studies included in this review, population characteristics were not always considered, especially the smoking status, periodontal status, and oral hygiene conditions, which can explain the inconsistent results to some extent. In fact, it is well-established that smoking (Al Bataineh et al., 2020), oral hygiene (Radaic and Kapila, 2021), and periodontal disease (Kumar et al., 2006) can influence the oral microbiome. It would not be sensible to evaluate the oral microbiome without considering the above factors. When the periodontal status of the participants was unknown, the results would be somewhat ambiguous as observations might have been due to the influence of periodontal disease (Corrêa et al., 2017). There were two studies performed in the periodontal examination of the participants; thus they were able to analyze the samples from periodontal healthy sites or individuals and confirm that the observed alterations of the oral microbiome were related with the SAD itself (Lopez-Oliva et al., 2018; Cheng et al., 2021).

Also, the effect of medications, especially the antibiotics, should be taken into consideration. Individuals with a history of antibiotics treatment in the last 2 weeks to 3 months were excluded in most studies (14/25), while in some studies the participants were undergoing treatment. Li et al. (2016) investigated the effect of prednisone on the oral microbiota in SS and found that *Lactobacillus* and *Streptococcus* were more affected by corticosteroids than the disease itself. RA therapy with potential antibacterial properties, such as methotrexate or hydroxychloroquine (Greenstein et al., 2007; Rolain et al., 2007), may also influence the oral microbiome. Therefore, future studies with treatment-naive individuals will be needed to clearly determine the role of oral microbiome in SADs.

There are many other confounding variables that should be considered. Decreased salivary secretion has a negative impact on the quantity of oral microorganisms, which can be seen in patients with SS. Thus, it is not clear whether the changed oral microbiome was caused by SS disease itself or the decreased salivary secretion. Interestingly, Siddiqui et al. (2016) have evaluated the microbiome of saliva in patients with SS with normal salivation and suggested that SS can lead to oral microbial dysbiosis independently of oral dryness. van der Meulen et al. (2018a) found that SS disease status and salivary secretion rate contributed almost equally to the variation of bacterial composition (3.8 vs. 4.3%). While another study observed that the reduction of salivary secretion contributed more to the changes in oral microbiome in patients with SS than the disease itself (van der Meulen et al., 2018b).

From this review, we found that it was difficult to prove a causal link between the oral microbial dysbiosis and disease by investigating the established SADs patients. At-risk individuals with RA development were included in some studies and dysbiosis were identified in their oral microbiome, indicating these perturbations may be related to the RA initiation (Tong et al., 2019; Cheng et al., 2021; Kroese et al., 2021). Cheng et al. found that a higher relative abundance of *Porphyromonas gingivalis* preceded the onset of clinical arthritis, supporting the hypothesis that oral microbial dysbiosis may be a cause of RA initiation (Cheng et al., 2021). However, for SLE and SS the current data was not sufficient to determine whether oral microbial dysbiosis is the consequence or the cause of diseases. Thus, the prospective cohorts of at-risk individuals should be included in the future study to elucidate the mechanisms underlying the potential link between the oral microbial dysbiosis and SADs.

In this systematic review, 92% studies (23/25) relied on the 16S rRNA sequencing technology, which is cost-effective and efficient to detect alterations in bacterial populations. However, a major limitation of this method is that only a single region of the bacterial genome can be analyzed and it is difficult to distinguish the species when their 16S rRNA gene sequences have high similarities (Větrovský and Baldrian, 2013). The shotgun metagenomics approach can provide information on the taxonomic composition of the ecosystem but also on functional genes in the sample, displaying several advantages over the 16S amplicon method, such as more confident identification of bacterial species, increased detection of diversity, and prediction of genes (Ranjan et al., 2016; Durazzi et al., 2021). However, it has been employed only in the two studies to investigate the oral microbiome of patients with RA (Zhang et al., 2015; Cheng et al., 2021). The changes in functional capability in the oral microbiome of patients with RA have been identified, although the actual function gene expression could not be determined by such a method. Besides the genomics, to the best of our knowledge, there was one study conducted by Konig et al. (2016) who analyzed the subgingival microbiome of patients with periodontitis using proteomic techniques and found that the citrullinome in periodontitis mirrored patterns of hypercitrullination observed in the rheumatoid joint. Periodontal pathogen *Aggregatibacter actinomycetemcomitans* has been identified as a candidate bacterial trigger of autoimmunity in RA. More proteomics, transcriptomics, and metabolomics technologies should be used for future studies and may provide a better understanding of the mechanisms underlying the association between oral microbiome and SADs.

In addition to RA, SLE, and SS, there are also other SADs not covered by this review, and few studies have investigated their oral microbiome. To the best of our knowledge, there was one study carried out by Zorba et al., who analyzed the smear samples from oral lesions of patients with pemphigus vulgaris (PV) using 16S rRNA sequencing and found that *Fusobacterium nucleatum* was the most dominant species (Zorba et al., 2021). In the future, high-throughput analysis could be used more widely to study the oral microbiome of other SADs.

## Conclusion

In this article, we presented a systematic review of literature that is focused on the big data analysis of oral microbiome of SADs patients. Oral microbial dysbiosis has been identified in all the SADs included in our review, by detecting the alterations in microbial composition and populations, as well as the function capabilities. Most dysbiosis features were different between studies, which could be due to a lack of standardized study methodology for each study, from the inclusion criteria, sample type, sequencing platform, referred database, to downstream analysis pipeline and cutoff. Besides the genomics, transcriptomics, proteomics and metabolomics technology should be used to investigate the oral microbiome of SADs patients and also the at-risk individuals of disease development, which may provide us with a better understanding of the etiology of SADs and promote the development of the novel therapies.

## Data Availability Statement

The original contributions presented in the study are included in the article/supplementary material, further inquiries can be directed to the corresponding author/s.

## Author Contributions

LG: conceptualization, methodology, validation, formal analysis, data curation, and writing. ZC: conceptualization, methodology, validation, formal analysis, data curation, writing, and funding. CB and QS: conceptualization, methodology, and writing. FZ: conceptualization, methodology, validation, writing, and funding. XC: conceptualization, writing, supervision, and administration. All authors contributed to the article and approved the submitted version.

## Funding

This study was supported by the Fundamental Research Funds for the Zhejiang Provincial Universities (2021XZZX033). ZC was supported by Young Elite Scientist Support Program by CSA (2020PYRC001). Support was also provided by the NSFC (82001048).

## Conflict of Interest

The authors declare that the research was conducted in the absence of any commercial or financial relationships that could be construed as a potential conflict of interest.

## Publisher's Note

All claims expressed in this article are solely those of the authors and do not necessarily represent those of their affiliated organizations, or those of the publisher, the editors and the reviewers. Any product that may be evaluated in this article, or claim that may be made by its manufacturer, is not guaranteed or endorsed by the publisher.
